# Shared immunological pathways in rheumatoid arthritis-related interstitial lung disease

**DOI:** 10.3389/fimmu.2025.1630729

**Published:** 2025-10-01

**Authors:** Jingyi Xu, Changhong Li, Jinxia Zhao, Rong Mu

**Affiliations:** Department of Rheumatology and Immunology, Peking University Third Hospital, Beijing, China

**Keywords:** rheumatoid arthritis, interstitial lung disease, pathogenesis, signaling pathway, targeted therapy

## Abstract

Interstitial lung disease (ILD) is a significant extra-articular complication of rheumatoid arthritis (RA), characterized by high prevalence and mortality rates. Although advancements have been made in understanding its potential mechanisms, the pathogenesis of RA-associated ILD remains incompletely understood. Recent research has shed light on roles of various disease-related signaling pathways, including TGF-β/SMAD, JAK/STAT, PI3K–Akt, Wnt/β-catenin, and NF-κB, which are implicated in development of both RA and lung fibrosis. These shared pathways, which drive inflammatory cytokine production and fibroblast proliferation, offer promising opportunities for therapeutic intervention, including pathway-specific inhibition and drug repurposing. Furthermore, the growing identification of potential biomarkers for early detection and severity assessment in RA-ILD patients holds promise for improving clinical management and guiding treatment strategies. Current treatments fall short in effectively halting the progression of lung fibrosis. This highlights the potential of advancements in signaling pathways and targeted therapies as promising alternatives with significant opportunities for improvement.

## 1 Introduction

Rheumatoid arthritis (RA) is a chronic systemic autoimmune disease characterized by symmetric polyarthritis which can cause irreversible joint damage and significant disability (1). In addition to the common articular involvement, extra-articular manifestations can also occur in RA patients, contributing to overall morbidity and mortality (2). The lungs, in particular, are frequently affected in individuals with RA experiencing lung-related issues (3). The disease can affect the lung’s interstitium, airways, and pleurae (4). Among these, interstitial lung disease (ILD) stands out due to its potential to cause irreversible pulmonary fibrosis, which can significantly worsen the prognosis for those affected, underscoring the importance of comprehensive care in managing RA (5). The histological features of RA-ILD can be categorized into usual interstitial pneumonia (UIP), nonspecific interstitial pneumonia (NSIP), organizing pneumonia (OP), desquamative interstitial pneumonia (DIP), and lymphocytic interstitial pneumonia (LIP) (6). Different from most forms of connective-tissue-associated ILD, UIP is more prevalent than NSIP, occurring in up to 61% of RA-ILD patients (7, 8). It shows poor responses to both glucocorticoids and immunosuppression (9). Therefore, patients with progressive fibrosing phenotypes are restricted to antifibrotic drugs, which often leads to high mortality (10).

Knowledge regarding mechanisms of RA-ILD has been greatly improved in recent years, as accumulated experimental evidence allows more detailed elucidation of potential molecular mechanisms and immunological pathways (4, 11, 12). However, many pathophysiological connections remain unclear. Based on knowledge of current literature, the central need in this area is for early recognition to prevent disease progression and effective therapeutic alternatives to reverse the condition (13, 14). However, this requires clear insights into pathogenesis and a better understanding of shared pathways involved. This review provides an overview of the key mechanisms in the pathogenesis of ILD in RA, as well as the advances in clinical screening and targeted therapies, highlighting areas of shared immunological pathways and ongoing uncertainties in related therapeutic opportunities.

## 2 Pathogenesis

The pathogenesis of RA-ILD usually involves the participation of multiple risk factors. Genetic predisposition has been implicated in the development of fibrosis and susceptibility to RA-ILD (15). Continuous epithelial damage, cellular interactions, and the interplay between the various inflammatory components could lead to the activation of fibroblasts and extracellular matrix deposition, which probably determines the progression of pulmonary fibrosis (4). During these processes, multiple signaling transduction pathways, including TGF-β/SMAD, JAK/STAT, PI3K–Akt, Wnt/β-catenin, and NF-κB signaling pathways were activated, triggering biological effects of cell proliferation and protein synthesis (16).

### 2.1 Genetic predisposition and risk factors

**Figure 1:**
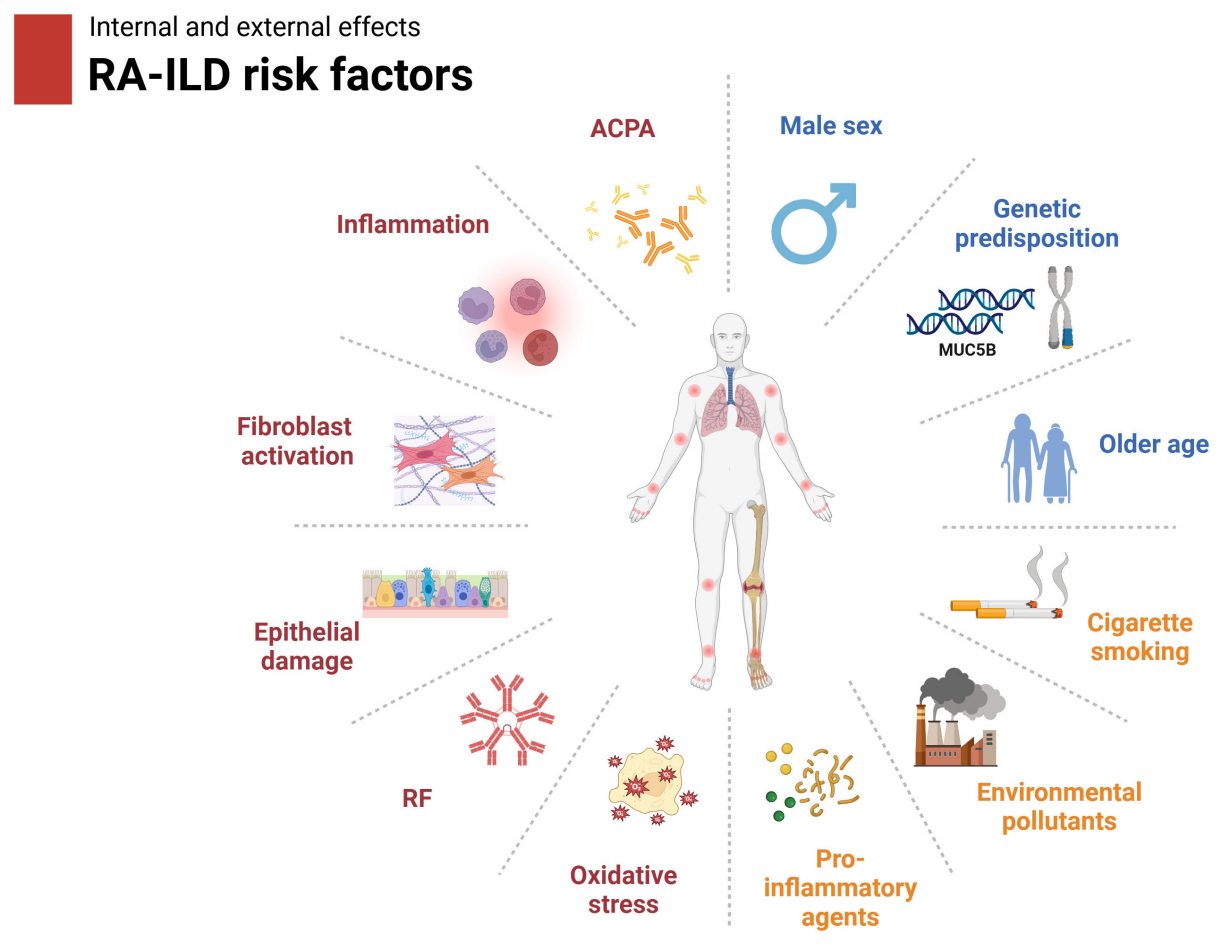
Summary of risk factors related to the pathophysiology of RA-ILD. Both endogenous and exogenous risk factors can contribute to the development of RA-ILD. Individuals with a genetic predisposition, especially older males, are at higher risk of developing RA-ILD. Environmental exposure to pollutants, cigarette smoking, and pro-inflammatory agents are also relevant. Internal dysregulations include epithelial damage, fibroblast activation, inflammation, oxidative stress, ACPA, and RF. RF, rheumatoid factor; ACPA, anti-cyclic citrullinated peptides antibody.

Potential risk factors related to the pathogenesis of RA-ILD are summarized in **Figure 1**. Both endogenous and exogenous risk factors can contribute to RA-ILD. Previous genetic studies have identified one specific variant of MUC5B promoter that serves as a strong indicator of increased risk of IPF (17). Specifically, this variant has been suggested to be associated with RA-ILD patients of the UIP pattern (15). For genes concerned with telomere maintenance, one study of whole-exome sequencing exhibited higher mutation frequency of TERT, PARN and RTEL1 in RA-ILD, which was previously related to familial pulmonary fibrosis (18). Patients with a TERT, RTEL1 or PARN mutation have shorter telomeres compared with control groups, indicating that premature senescence may play a role in fibrosis and lung remodeling in RA patients (19). Genetic predisposition of HLA genes to ILD in RA patients has been studied. A lower risk of ILD was found in RA population with shared epitope of HLA-DRB1 in a Japanese cohort (20). Differently, HLA-DRB1*15 and HLA-DRB1*16 from the HLA-DR2 alleles were correlated with a higher risk of ILD (21). Additionally, another genetic variant at RPA3-UMAD1 was identified in Japanese individuals with RA-ILD (22).

Currently, risk factors associated with RA-ILD include genetic variations, older age, male gender, race, and cigarette smoking (23, 24). The abnormal immune response induced by chemical components of cigarettes may underlie the potential mechanism contributing to ILD formation (25). Meanwhile, autoantibodies, including RF and ACPA have been demonstrated to be associated with RA-ILD progression (26). Continuous exposure to risk factors contributes to epithelial deterioration and activation of immune cells, leading to the excessive accumulation of extracellular matrix in the lung (27).

### 2.2 Epithelial damage, inflammation, and fibroblast activation

**Figure 2:**
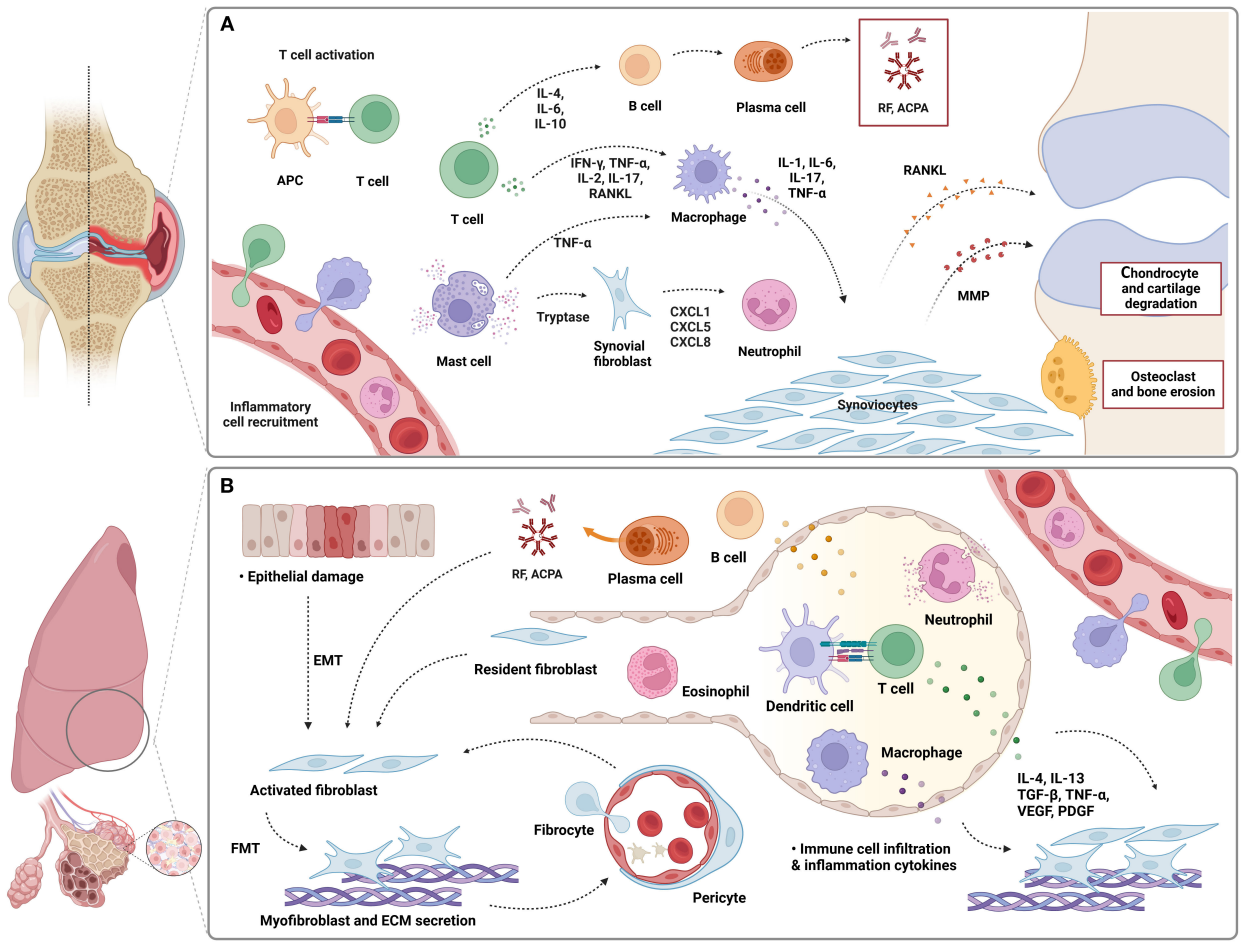
Possible mechanisms of rheumatoid arthritis **(A)** and related interstitial lung disease **(B)**. Pathogenesis of parenchymal lung involvement in RA includes participation and activation of diverse immune cells such as T cells, B cells, macrophages, neutrophils, and eosinophils. They contribute to joint damage and pulmonary fibrotic responses through the secretion of pro-inflammatory cytokines. Fibroblasts both at affected sites and circulation are involved in the self-sustaining of lung fibrosis. APC, antigen-presenting cells; IFN-γ, interferon-γ; TNF-α, tumor necrosis factor; CXCL, C-X-C motif ligand; MMP, matrix metalloproteinase; RANKL, receptor activator of nuclear factor kappa-B ligand; EMT, epithelial-mesenchymal transition; FMT, fibroblast-to-myofibroblast transition; IL, interleukin; TGF-β, transforming growth factor-β; ECM, extracellular matrix; VEGF, vascular endothelial growth factor; PDGF, platelet-derived growth factor.

Most theories propose the mucosal sites, including airway, gastrointestinal, or genitourinary tract, may serve as potential origins of inflammation (28). Among them, the airway mucosa plays a critical role in the initiation of RA-related autoimmunity, as it is the site where the predisposing environmental factors interact with the host, resulting in local injury and triggering an inflammatory response (28). Constant alveolar epithelial injury and immune stimulation induced by RA trigger inflammatory responses in pulmonary tissue, resulting in infiltration of inflammatory cells into the interstitial and alveolar airspaces (24). Their activation and interactions, along with the participation of a wide profile of cytokines, are critical to the generation of abnormal fibroproliferative responses, as summarized in **Figure 2**.

One critical step in the development of interstitial fibrosis is related to activated fibroblasts and myofibroblasts resident within the lung, as well as recruitment of progenitor cells from blood circulation (29). Evidence from animal models suggested the localization and differentiation of circulating progenitor cells into fibrocytes, including mesenchymal stem cells and a monocyte lineage, contributing to the accumulation of extracellular matrix proteins and tissue fibrosis (30). Additionally, the epithelial cells of lung tissue have been shown to participate in the profibrotic process via a mechanism known as epithelial–mesenchymal-transdifferentiation (EMT), during which they acquire mesenchymal characteristics and behaviors (31). This transition is upregulated in animal models of RA-ILD triggered by signals from the microenvironment, resulting in an increased profibrotic mesenchymal cell population (32, 33). Furthermore, fibroblast-myofibroblast transition (FMT) will then accelerate the process of fibrosis by translating activated fibroblasts into myofibroblasts, establishing a self-sustaining cycle (34).

## 3 Activation of signaling pathways involved in RA and ILD

### 3.1 TGF-β/SMAD signaling pathway

**Figure 3:**
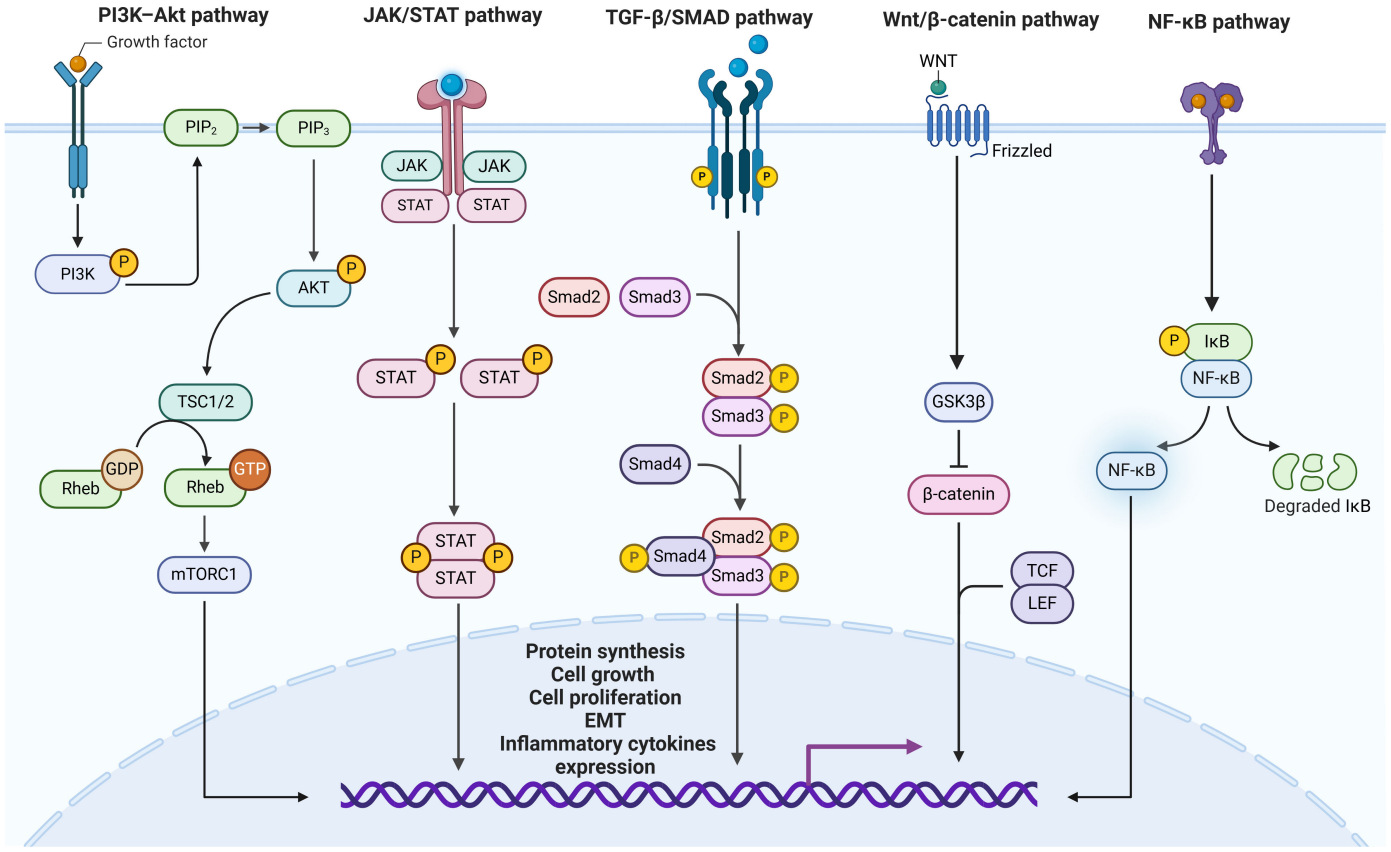
Model of shared mechanistic pathways involved in both RA and ILD. Activation of TGF-β/SMAD, JAK/STAT, PI3K–Akt, Wnt/β-catenin, and NF-κB signaling pathways and resultant downstream reactions contribute to multiple biological process. Improved understanding of their roles in RA-ILD pathogenesis offers promising therapeutic targets. PIP, phosphatidylinositol bisphosphate; mTORC1, mechanistic target of rapamycin complex 1; STAT, signal transducer and activator of transcription; TCF/LEF, T cell factor/lymphoid enhancer-binding factor; GSK3β, glycogen synthase kinase 3 beta; EMT, epithelial-mesenchymal transition.

TGF-β signaling is an important pathway in fibrosis mechanism regulating response of tissue repair (35). Pleiotropic effects are mediated via three different types of TGF-β receptors, including TGFβRI, TGFβRII, and TGFβRIII (36). Dysregulation of TGF-β signaling has been implicated in disease development (37). In particular, over-activation of TGFβRI and TGFβRII can induce excessive collagen accumulation and progressive fibrosis in pulmonary tissues (38, 39). Recent studies have revealed that the activation of TGF-β-SMAD2/3 signaling promotes EMT in lung fibrosis through establishing an animal model of RA-ILD mice (32). Furthermore, in another RA mouse model, pulmonary fibrosis can be reduced by inhibiting pathway of TGF-β/Smads signaling, during which proliferation and migration of RA synovial fibroblasts were also restricted (40). Insights from mechanism studies of autophagy also indicated that a decreased number of activated myofibroblasts can be induced by enhancing autophagy activity via inhibition of TGF-β1-Smad3/ERK/P38 signaling pathway (41). TGF-β inhibition therapy carries great expectations and are under investigation to improve safety and efficacy (42) (**Figure 3**).

### 3.2 JAK/STAT signaling pathway

JAK/STAT pathway can be activated by various cytokines and growth factors associated with fibrosis and inflammation, such as IL-11, IL-6, TGF-β1, and fibroblast growth factors (FGFs) (43, 44). It participates and mediates extensive biological processes, including cell differentiation and immune regulation, closely related to autoimmune disorders and fibrosis (45). The JAK/STAT signaling system is widely recognized as a key molecular mechanism in the etiology and development of RA, and it has emerged as a novel effective target in RA therapeutic strategies (46, 47). Given its involvement in several fibrotic disorders, there has been an interest in exploring its potential roles in ILDs (48). Several studies revealed that JAK2/STAT3 is the most common subtype in ILD, which participates in intercellular signal transduction in response to the stimulation of cytokines and growth factors (49, 50). RNA sequencing and immunohistostaining have provided evidence that JAK2 serves as a regulator and intermediator of TGF-β-mediated lung fibroblast responses in the development of RA-ILD with a usual interstitial pneumonia (RA-UIP) and IPF (51). In contrast, their findings suggested STAT5 as the downstream target in transcription, necessitating further investigation for clarification (51).

### 3.3 PI3K–Akt signaling pathway

The signaling pathway of PI3K-Akt has a great effect on cell activation and migration (52). In patients with ILD disease, it has been demonstrated to be aberrantly upregulated, contributing to tissue repair and disease progression (53). Enhanced activity of PI3K/AKT signaling pathway is related to synovial inflammation and osteoclasts proliferation in RA pathogenesis (54). PI3Kγ affects innate immunity of antigen-induced arthritis in the early phase by regulating downstream signal molecules, during which activated macrophage and neutrophil infiltrate into the joint (55). Research on PI3Kγ has also indicated that it may regulate synovial inflammation via fibroblasts, resulting in cartilage destruction (56). Impact of PI3K/AKT signaling pathway on osteoclast differentiation and generation may also contribute to aggravation of RA (56). Consequently, PI3K represents a viable therapeutic target for RA due to its central role in mediating inflammatory responses and bone destruction. Aberrant activation of PI3K/AKT has also been observed in IPF patients and demonstrated to play a central role in pathogenesis of the disease, which was considered as an effective therapeutic target against lung fibrosis (57, 58). Our research team has recently found that the CXCL16/CXCR axis can facilitate pulmonary fibrosis by activating PI3K/AKT/FOXO3a signaling pathway in human pulmonary fibroblasts, stimulating cell proliferation and collagen production (59). Another study elucidated upregulation of PI3K-Akt signaling pathways in RA-ILD, based on network pharmacological and metabolic perspectives (60). Pharmacological inhibitors targeting PI3K/AKT pathway are presently being investigated in clinical trials for IPF treatment, demonstrating promising potential as innovative anti-fibrotic therapeutics (57).

### 3.4 Wnt/β-catenin signaling pathway

Research on Wnt pathway in RA has suggested that it plays a role in synovial cell proliferation and bone homeostasis, promoting synovial hyperplasia and contributing to joint damage (61, 62). The Wnt/β-catenin signaling pathway was significantly activated in IPF, with participation of a sequence of related ligands and receptors (63). Molecules involved in Wnt pathway, such as cyclin-D1 and matrilysin, were found to be over-expressed in lesions of pulmonary fibrosis (64). One study focusing on M2 macrophages indicated that inhibition against Wnt/β-catenin signaling pathway may suppress myofibroblast differentiation and pulmonary fibrosis (65), suggesting that repurposing Wnt-targeting agents could hold promise for RA-ILD. Since shared Wnt signaling was involved in ILD and RA, a recent study investigated the clinical significance of a representative ligand Wnt5 in RA-ILD: Elevated levels of plasma Wnt5a protein were observed in patients with RA-ILD, especially in patients with UIP pattern (66). Accordingly, evaluation of circulating Wnt5a may provide a potential candidate biomarker for early identification and progression assessment of RA-ILD (66). Furthermore, Wnt5a inhibitors or receptor antagonists could be explored to mitigate fibrotic progression in RA patients.

### 3.5 NF-κB signaling pathway

NF-κB signaling pathway is widely acknowledged for its role in the activation and differentiation of various immune cells, closely related to human inflammatory response (67). Dysregulated activation of NF-κB promotes chronic inflammation and excessive proliferation of fibroblast-like synoviocytes in RA (68, 69). Activated NF-κB members in the synovial tissue of RA patients can induce activation and differentiation of immune cells as well as the upregulation of various pro-inflammatory cytokines such as TNF-α, IFN-γ and IL-17, contributing to the aggregation of RA progression (70). It was also found in an animal model of lung fibrosis that fibroblast-to-myofibroblast differentiation, which contributes to aberrantly activated lung myofibroblasts and extracellular matrix accumulation, is dependent on the activation of NF-κB signaling (71). Moreover, one study supports IL-17A of its direct role in stimulating positive responses of lung fibroblasts proliferation and extracellular matrix accumulation in RA-ILD pathogenesis via signaling mediated by NF-κB (72). Therapeutically, this underscores why NF-κB inhibition remains a key strategy in RA management, while new approaches are being explored to directly target this pathway and mitigate lung fibrosis.

## 4 Biomarkers of clinical identification and monitoring

Biomarkers correlated with RA-ILD development serve as useful tools for early screening and intervention guidance. Autoantibodies, cytokines, and novel biomarkers of other properties provide valuable guidance in screening and prediction of disease progression. (**Table 1**) As a group of auto-antibodies widely used for early RA diagnosis, ACPA is also identified as a risk factor and strong predictor of RA-ILD (73, 74). It may become a useful serological biomarker to predict the presence and progression of lung fibrosis in RA patients (75, 76). Additionally, an elevated level of RF is also suggested to be associated with clinically evident and subclinical RA-ILD risk (75, 77). Recent studies indicate that the autoantibodies targeting citrullinated Hsp90α and Hsp90β in patients with RA-ILD exhibit moderate sensitivity and great specificity in predicting RA-ILD progression (78).

**Table 1:** Potential serum biomarkers for RA-ILD.

Biomarkers	Property	Potential value in clinical evaluation	References
ACPA	Autoantibody	Elevated levels associated with clinically evident and subclinical RA-ILD; specific ACPA improve RA-ILD prediction	(75, 76)
Rheumatoid factors	Autoantibody	Elevated levels associated with clinically evident and subclinical RA-ILD; a potentially useful prognostic marker of disease activity and mortality risk	(75, 77)
Anti-citrullinated Hsp90α/β	Autoantibody	Distinguishing RA-ILD patients from RA without ILD, or MCTD, IPF patients; useful biomarkers of high specificity and moderate sensitivity	(78)
MMP-7	Extracellular remodeling proteinase	Elevated in RA patients with organ-specific manifestation of ILD; contribute to the identification of RA-ILD during subclinical stage; negatively correlated with PFT variables	(75, 80, 160)
CXCL10	Cytokine	Elevated in RA patients with organ-specific manifestation of ILD; a sensitive biomarker of disease activity and predictor of RA-ILD	(80, 161)
IL-13	Cytokine	Detecting the severity of ILD in RA patients	(162)
IL-18	Cytokine	Associated with RA-ILD diagnosis and progression of lung disease	(163)
CCL18	Cytokine	Higher concentrations in patients with RA-ILD	(164, 165)
PARC	Cytokine	Contribute to identification of RA-ILD during subclinical stage	(75)
sPD-L1	Soluble checkpoint inhibitor	A predictive marker for detecting the presence of ILD	(166)
LOXL2	Oxidative stress marker	Diagnostic value for RA patients with ILD duration less than 3 months	(167)
KL-6	Lung epithelium-derived protein	Correlated with HRCT results and PFT variables; a circulating marker for disease diagnosis and progression	(160, 165, 168)
YKL-40	Lung epithelium-derived protein	A useful biomarker in diagnosis and severity evaluation	(161, 169)
Periostin	Matrix protein	A potential biomarker for diagnosis and fibrosis evaluation	(170)
SP-D	Lung epithelium-derived protein	Contribute to the identification of RA-ILD during subclinical stage; negatively correlated with PFT variables	(75, 160)
Uric acid	Metabolite	A diagnostic biomarker in RA-ILD, particularly in RA-UIP	(85)
Hsa-miR-214-5p and has-miR-7-5p	MicroRNA	Useful biomarkers for diagnosis	(88)

ACPA, anti-citrullinated peptide antibodies; MCTD, mixed connective tissue disease; MMP-7, matrix metalloproteinases-7; PFT, Pulmonary Function Test; CXCL, C-X-C motif chemokine ligand; IL, Interleukin; CCL, chemoattractant cytokine ligand; PARC, pulmonary and activation-regulated chemokine; sPD-L1, soluble programmed death ligand-1; LOXL2, lysyl oxidase like 2; KL-6, Krebs von den Lungen-6; YKL-40, Chitinase-3-Like protein; SP-D, surfactant associated protein D.

Matrix metalloproteinases (MMPs) are enzymes involved in extracellular matrix degradation and tissue remodeling (79). The serum concentration of MMP-7 and CXCL10 was elevated in patients with RA-ILD compared with RA without ILD (80). Meanwhile, another research manifested a profile of biomarkers relating to lung disease, including MMP-7, activation-regulation chemokines, and surfactant protein D, which may be conducive to early diagnosis during the subclinical stage (81, 82). One study compared the profiling of serum proteins and demonstrated that lysyl oxidase-like 2 (LOXL2) levels were significantly increased in RA individuals, especially in those patients with ILD duration less than three months (81). Krebs von den Lungen-6 (KL-6) is a mucin-like glycoprotein associated with pulmonary fibrosis and is also a promising indicator for RA-ILD diagnosis and severity assessment (83, 84).

It is noteworthy that, in addition to protein, other chemicals can also provide valuable information in disease monitoring. Evidence from the latest studies indicated uric acid can become a useful diagnostic biomarker in RA-UIP (85–87). Interestingly, a noticeable positive connection between microRNA, especially Hsa-miR-214-5p and has-miR-7-5p, and RA-ILD was also discovered recently, which exhibits prospectives in future studies (88).

## 5 Existing therapies and safety concerns

According to treatment algorithms for RA-ILD recently built by the ERS/EULAR clinical practice guidelines, pirfenidone is recommended for patients with UIP pattern and combination of immunosuppressive treatment is suggested in patients diagnosed with severe or progressive ILD (89). Moreover, nintedanib or combination of immunosuppressant and nintedanib is recommended for individuals with progressive pulmonary fibrosis (89). For RA-ILD with active arthritis, managing RA to target is of great importance. Currently, drug therapy for RA, such as conventional DMARDs (cDMARDS) and biologic DMARDs (bDMARDs) is widely recognized, while such effects may be balanced by higher risks of drug-related pulmonary disease (90). Until now, no evidence from randomized controlled trials (RCTs) has been found to optimize RA-ILD patient management. (**Table 2**).

**Table 2:** Recent clinical trials of pharmacological interventions in RA-ILD.

Intervention	Trial name	Phase	Population	Primary ending point	Current status
Pirfenidone	TRAIL1(155)	II	RA-ILD	Decline in FVC% from a baseline of 10% or more or death	Completed
DMARDs & pirfenidone	NCT04928586	IV	CTD-ILD	Change in FVC and DLCo	Active, not recruiting
Pirfenidone & glucocorticoid & immunosuppressant	NCT05505409	IV	CTD-ILD	Change in FVC%	Recruiting
Abatacept	APRIL	II	RA-ILD	Change of FVC	Unknown status
Nintedanib	NCT05503030	Observational study	CTD-ILD	Change in FVC and dyspnoea symptom score	Recruiting
Tofacitinib	RAILDTo	II	RA-ILD	Incidence and severity of adverse events	Recruiting
Tofacitinib vs Methotrexate	PULMORA	IV	RA-ILD	Change in total score by HRCT	Terminated
Genakumab injection	NCT06189495	II	RA-ILD	Change in FVC, DLCo, PGA, and adverse events	Not yet recruiting
N-acetylcysteine	NCT01424033	II, III	CTD-ILD	Pulmonary Function Tests	Terminated

DMARDs, disease-modifying anti-rheumatic drugs; CTD-ILD, connective tissue disease-associated interstitial lung disease; FVC, forced vital capacity; DLCo, diffusing capacity for carbon monoxide; HRCT, high resolution CT; PGA, patient global assessment.

### 5.1 Methotrexate

Methotrexate (MTX) is the anchor DMARD in treating RA patients (91). However, despite its significant efficacy and tolerability, a range of potential side effects, especially pulmonary toxicity, cannot be overlooked (92). It is conventionally believed that MTX is associated with a higher risk of adverse respiratory events, including fibrotic ILD (93). According to some retrospective studies, MTX is correlated with the occurrence or exacerbation of ILD, constituting as much as 7.6% of the ILD incidence (94). On the other hand, the direct causality of MTX and ILD remains undefined. According to one meta-analysis in 2021, MTX treatment is not associated with a higher risk of RA-ILD among RA patients (95). One Korean prospective cohort of RA-ILD patients also exhibits similar results, indicating that MTX does not contribute to the increased risk of ILD progression (96). Even better, recent evidence from a multi-center prospective cohort study suggested MTX can reduce the onset of ILD, supporting the beneficial role of MTX in RA-ILD (97). In addition, recently research has suggested that use of MTX is related to improvement and longer survival among RA-ILD patients (98). By contrast, prospective studies can avoid time-window bias, thereby providing stronger evidence of causality. Therefore, traditional view of correlations between MTX and ILD progression has been challenged.

### 5.2 Biologic drugs

#### 5.2.1 TNF inhibitors

Currently, no guidelines have been established when deciding biologic agents or targeted therapies in RA-ILD patients. The efficiency of anti-TNF agents in alleviating articular and synovial damage is supported by previous studies, while their role in stabilizing RA-ILD remains controversial (99, 100). Numerous studies have demonstrated that no significant beneficial effects were seen between patients with RA-ILD treated with anti-TNF agents and other therapies (101, 102). Moreover, it is also suggested that a higher prevalence of ILD among RA patients is associated with TNF-inhibiting treatment (103). Results of one cohort study illustrated a lower survival rate of RA-ILD patients using TNFi as the first-line therapy by comparing their 5-year mortality rates with groups taking rituximab (RTX) as the first-line treatment (104). Another prospective case-control study has also demonstrated the exacerbation of ILD in cases treated with TNFi compared with non-TNFi biologic agents including tocilizumab and abatacept. Additionally, TNFi is indicated to be related to a higher incidence or development of ILD according to their finding (105). Therefore, given the current understanding, increased focus is necessary to ensure the efficacy and safety of biological therapy in RA-ILD patients (106).

#### 5.2.2 Rituximab, abatacept and tocilizumab

Other bDMARDs, such as RTX and abatacept are more advised to be considered as alternatives in treating RA-ILD. According to a ten-year retrospective observational cohort conducted among 700 RA patients, RTX can serve as an acceptable therapeutic option with efficacy in stabilizing RA-ILD (107). Significant improvement in %-predicted FVC was found in RA patients with RA-ILD under rituximab treatment (108). Meanwhile, evidence from systemic review suggested a significant lower worsening rate of ILD under therapy of abatacept in RA-ILD compared with TNF inhibitors, which therefore would become a desirable alternative for RA-ILD patients (109). Moreover, one recent cohort study on tocilizumab declared that positive effects were shown in most RA-ILD patients during a 6-month period by monitoring biomarkers of both MMP-3 and KL-6. However, it is noteworthy that condition of ILD can be worsened by respiratory infection after one year of tocilizumab therapy (110). Currently, ongoing clinical trials on RA-ILD are summarized in **Table 2**. Due to the absence of prospective randomized controlled studies for further confirmation, more detailed therapeutic guidelines and recommendations are yet to be established.

## 6 Pathway-targeted therapy

### 6.1 Targeting JAK/STAT signaling pathway

JAK inhibitors, such as tofacitinib and baricitinib, are novel effective therapies in the treatment of RA (111, 112). Based on data from the most recent studies, JAKi shows great tolerance in RA-ILD patients and comparable efficacy and safety to bDMARDs including RTX and abatacept (113). Since they can alleviate the joint damage of RA patients by controlling serum inflammatory cytokines and exerting anti-fibrotic effects, they may also lead to clinical improvement in RA patients with ILD (114). One retrospective study examined the effectiveness of JAK inhibitors in patients with RA-ILD, among whom 83.9% of patients showed clinical stability or improvement after 18 months of treatment (115). Meanwhile, studies in 2019 demonstrated the pathogenic role of IL-17A, which is a T helper 17 cytokine, involved in the pathogenesis of RA and pulmonary fibrosis. Their observations have supported the fibrogenic response in RA-ILD induced by IL-17A, which can be significantly alleviated with the inhibition of JAK2 (72).

Despite limited knowledge from clinical trials, tofacitinib, a JAK inhibitor, represents an effective anti-inflammatory therapeutic option for RA patients with lung involvement. Case reports have suggested that tofacitinib contributes to the stabilization of ILD in RA patients (116, 117). Recently, a large retrospective cohort consisting of 28,559 RA patients treated with tofacitinib showed a much lower incidence of ILD compared with patients treated with other bDMARDs, suggesting their significance in reducing risk of ILD in RA (118). Baricitinib, a JAK1/JAK2 inhibitor, was demonstrated to show promising effects in RA patients with interstitial lung involvement. In patients under baricitinib therapy, significant improvement in lung diffusion index and reduction of biomarkers was observed (114). Additionally, administration of JAK1 selective inhibitor upadacitinib in RA-ILD patients who were resistant to MTX and TNFi has shown improved outcomes, as reported by retrospective studies as well as individual cases (119, 120).

However, safety concerns with JAKi are also noteworthy. Black box warnings have already been raised by FDA, attaching importance to increased risks of cardiovascular diseases, infection and cancer, inducing regulatory restrictions (121). According to evidence from clinical trials, adverse outcomes are more commonly observed in the elderly, or individuals with baseline cardiovascular risks, inflammation status and disease activity (122). Further investigations into JAK inhibitors as novel therapeutic options to treat RA-ILD patients as well as their safety profile will be of great significance.

### 6.2 Other promising pathway-related therapeutic options

Since shared molecular pathways exist in both RA and ILD, key molecules involved in signaling transduction may provide promising targets for developing novel therapeutic strategies for RA-ILD patients. Pan-Phosphodiesterase (PDE) inhibitors were found to exert a potent effect in anti-remodeling via inhibiting the target of the TGF-β1 signaling pathway against lung fibrosis (123). Given that they possess potent anti-inflammatory activities by modulating various immune cell functions, they are also considered a potential therapeutic strategy for RA (124, 125). Additionally, selective PDE4 inhibitors may also inhibiting the inflammatory reaction of RA-ILD via inhibition of transcription factor NF-κB in PBMC (126). However, their effectiveness in RA-ILD lacks further evidence from experiments and clinical trials. ACPA has been associated with RA progression and RA-ILD exacerbation and peptidylarginine deiminase 2 (PAD2) is an enzyme that catalyzes protein citrullination. Increased levels of PAD2 were detected in lung homogenates in RA-ILD subjects. Syndecan-2 was associated with internalization and degradation of TGF-β receptor in alveolar epithelial cells and was determined to exert antifibrotic effects on RA-ILD fibroblasts by regulating PAD2 (55, 127). Evidence from mouse model confirmed that PAD2 can be downregulated by Syndecan-2 through PI3K/Akt signaling in lung fibroblasts. Matrix metalloproteinase-9 (MMP9) is known as a proteolytic enzyme that promotes the formation of synovial pannus and cartilage destruction in RA. Anti-MMP9 antibody andecaliximab can alleviate pulmonary fibrosis via TGF-β1-induced Smad2 phosphorylation blockade in mouse model, exhibiting beneficial effects in patients with higher expression of type 1 IFNs in particular (128). Its safety and effectiveness in RA patients have been assessed through a Phase1b clinical trial (129).

Meanwhile, target analysis and molecular docking to explain the effectiveness of triptolide and tripterine in CTD-ILD intervention suggest PI3K-Akt was identified as one of the main signaling pathways in molecular mechanism (130, 131). Findings demonstrated that it participates in regulation of fibrotic extracellular matrix (ECM) remodeling and therefore exhibits therapeutic value against pulmonary fibrosis (132). Paroxetine, a GRK2 inhibitor, was recently reported to suppress the activation of immune cells by inhibiting PI3K-AKT-mTOR pathway in patients with RA (133). Since GRK2 is also involved in TGF-β1-induced activation of lung fibroblasts, paroxetine is demonstrated to produce therapeutic effects in pulmonary fibrosis (134). Further clinical trials of patient cohort will be warranted. Previous opinions have considered PTEN as a critical tumor suppressor protein and also a crucial protector against alveolar epithelial injury and pulmonary fibrosis by controlling the PI3K/AKT signaling pathway (135, 136). Current findings on PTEN in RA pathogenesis indicated that its aberrant function contributes to fibroblast-like synoviocyte activation and bone destruction, which offers new opportunities to discover potential therapeutic targets (137).

Exosome component 4 (EXOSC4), a component of the RNA exosome complex, was identified to be a potential autoantigen for RA-ILD patients. Recent findings suggested that levels of EXOSC4 antibodies were found to be significantly increased in sera of RA-ILD patients and it may contribute to the pulmonary fibrotic process via activation of Wnt/β-catenin signaling pathway (138). Knockdown of EXOSC4 can inhibit the activity of Wnt/β-catenin pathway and regulate the proliferation and differentiation of ATII cells, which provide valuable insights into potential mechanisms and therapy. Currently, it already serves as a therapeutic target in colorectal and ovarian cancers (139, 140). Meanwhile, since Wnt/β-catenin signaling pathway play a significant role in pulmonary fibrosis, other blockades of this pathway, including Calcaratarin D and Betulinic acid, were indicated to become novel antifibrotic agents for pulmonary fibrosis (141, 142).

Osthole obtained from plants was estimated to restrain the function of RA-fibroblast-like synoviocytes through inhibition of NF-κB signaling, exerting a therapeutic effect in RA development. Meanwhile, by controlling accumulation and polarization of M2 macrophages in pulmonary interstitial fibrosis, it may also exhibit beneficial properties in RA patients with complications of ILD (143). Berberine has been reported with the potential to prevent RA and suppress inflammation through multiple signaling pathways, including Wnt/β-catenin, PI3K/Akt, and NF-κB (144). Its inhibitory effects to attenuate lung fibrosis through EMT and oxidative stress suppression as well as autophagy stimulation have also been confirmed by multiple relevant studies via animal models (145, 146). Further research and clinical trials on these pathway-related therapeutic targets exhibit great potential for improving the clinical outcomes of RA-ILD patients.

## 7 Antifibrotic management

Accumulating interest has been drawn to the application of antifibrotic medications against ILD progression, given the shared histological patterns and pathogenesis between IPF and RA-ILD. Pathways targeted by antifibrotic drugs, such as activation of TGF-β, WNT signal pathway and YAP/TAZ signal pathway, have become important strategies for fibrosis reduction (147). Novel therapeutic strategies focusing on the mechanism of fibrosis in RA-ILD, including nintedanib and pirfenidone, have recently been demonstrated to be promising in improving the outcomes of RA-ILD patients (148). Current viewpoints suggest the synergistic role of anti-fibrotic agents in treatment of RA-ILD combined with DMARDs.

Evidence from the INBUILD trial has indicated that in groups treated with nintedanib, the rate of FVC decline was slower, both in patients with or without background therapy of DMARDs and/or glucocorticoids (149). Furthermore, it is suggested that compared with other fibrotic patterns, patients with UIP patterns shown by HRCT are more likely to benefit from nintedanib administration (149). However, patients under high-dose of immunosuppressive treatment were excluded from INBUILD probably due to safety concerns such as infection risks and hepatotoxicity. So far, data on high-dose combinations remain limited (150). Hepatotoxicity is one of the common adverse effects. An increase of ALT level over three times of the upper limit was reported in 15.1% patients treated with nintedanib (151).

The potential effect of pirfenidone, another anti-fibrotic drug, was also being evaluated among RA-ILD patients. Similarly, studies recently have indicated their protective role against disease progression by slowing down FVC decline in RA-ILD patients (152). Pirfenidone can inhibit the transformation of fibroblasts into myofibroblasts and result in the reduction of critical cytokines involved in RA pathogenesis, including IL-6 and TNF-α (153, 154). While discontinuations have been reported in TRAIL1 due to adverse effects on gastrointestinal system (24.8%) (155). Other side effects include incidences of photosensitivity (12%) and rash (32%) in randomized trials (156). Further studies on antifibrotic drugs will be necessary to clarify their efficacy and safety in RA-ILD treatment.

As for recent progress in cell therapy, rapid clinical improvement was observed in cases using CD19-targeting CAR-T cell therapy in treatment of CTD-ILD refractory to traditional immunosuppressive therapies (157). Last but not least, development of drug delivery systems based on RNAs, oligonucleotides, exosomes, and stem cells against pulmonary fibrosis is also worth noticing (158). For instance, one study developed mesenchymal stem cell-derived extracellular vesicles to treat pulmonary fibrosis by targeting senescent alveolar epithelial cells (159).

## 8 Conclusions

This review summarized the latest advances in RA-ILD, addressing the unsolved issues with great significance to inform future directions. Refined knowledge of the mechanistic interplay suggested a potential linkage of RA and ILD pathogenesis, involving multiple contributing factors. Meanwhile, progress in understanding the convergence of immunological pathways will contribute to a shared paradigm in patient management with considerations for both articular damage and the risk of lung fibrosis. Evidence of clinical evaluation from biomarkers is meaningful to allow for early identification from subclinical conditions, thereby predicting disease progression and guiding algorithms of optimal management. Currently, targeted therapies for RA treatment and their safety profile are being considered within clinical trials, while data regarding their use in RA-ILD as well as other efficacious options against pulmonary fibrosis are still limited. An urgent need remains for more studies on pathway-targeted therapeutic strategies to search for underlying opportunities that improve the overall outcomes of RA-ILD patients. Future research should prioritize the clinical validation of novel targeted therapies and exploration of combination therapies that concurrently address inflammation and fibrosis. By integrating these approaches, we can advance toward more effective, personalized treatment strategies that transform the management paradigm of RA-ILD.
